# Acute medical missions by helicopter medical service (HEMS) to municipalities with different approach for primary care physicians

**DOI:** 10.1186/s12873-022-00655-z

**Published:** 2022-06-08

**Authors:** Dag Ståle Nystøyl, Øyvind Østerås, Steinar Hunskaar, Erik Zakariassen

**Affiliations:** 1 Department of Research, Norwegian Air Ambulance Foundation, Oslo, Norway; 2 Department of Global Public Health and Primary Care, Group for Health Services Research, University of Bergen, Bergen, Norway; 3 Department of Anaesthesia and Intensive Care, Haukeland University Hospital, Bergen, Norway; 4 National Centre for Emergency Primary Health Care, NORCE Norwegian Research Centre, Bergen, Norway

**Keywords:** Emergency medical services, Primary health care, Air ambulances, Norway, HEMS, General practitioners, After-hours care, Out-of-hours medical care

## Abstract

**Background:**

The prehospital emergency system in Norway involves out-of-hours (OOH) services with on-call physicians. Helicopter emergency medical service (HEMS) are used in cases of severe illness or trauma that require rapid transport and/or an anesthesiologist’s services. In recent years, on-call primary care physicians have been less available for call-outs in Norway, and HEMS may be requested for missions that could be adequately handled by on-call physicians. Here, we investigated how different availability of an on-call physician to attend emergency patients at site (call-out) impacted requests and use of HEMS.

**Methods:**

Our analysis included all acute medical missions in an urban and nearby rural OOH district, which had different approach regarding physician call-outs from the OOH service. For this prospective observational study, we used data from both HEMS and the OOH service from November 1^st^ 2017 until November 30^th^ 2018. Standard descriptive statistical analyses were used.

**Results:**

The rates of acute medical missions in the urban and rural OOH districts were similar (30 and 29 per 1000 inhabitants per year, respectively). The rate of HEMS requests was significantly higher in the rural OOH district than in the urban district (2.4 vs. 1.7 per 1000 inhabitants per year, respectively). Cardiac arrest and trauma were the major symptom categories in more than one half of the HEMS-attended patients, in both districts. Chest pain was the most frequent reason for an OOH call-out in the rural OOH district (21.1%). An estimated NACA score of 5–7 was found in 47.7% of HEMS patients from the urban district, in 40.0% of HEMS patients from the rural OOH district (*p* = 0.44), and 12.8% of patients attended by an on-call physician in the rural OOH district (*p* < 0.001). Advanced interventions were provided by an anesthesiologist to one-third of the patients attended by HEMS, of whom a majority had an NACA score of ≥ 5.

**Conclusions:**

HEMS use did not differ between the two compared areas, but the rate of HEMS requests was significantly higher in the rural OOH district. The threshold for HEMS use seems to be independent of on-call primary care physician involvement.

## Background

Different industrialized countries exhibit different organizations of prehospital emergency medical services [1]. The system is two-tied, with the specialized health service responsible for the EMCC and ambulances included HEMS, while municipalities are responsible for primary care OOH services with a on call physician [2]. Norwegian legislation requires that municipalities have at least one physician on-call 24/7, with the ability to call-out when needed in emergencies [2]. Ambulances are manned by emergency medical technicians (EMT) who have a minimum of two years in high school and two years of apprenticeship, while helicopter emergency medical service (HEMS) is manned by an anesthesiologist and a nurse or paramedic. The on-call physicians in the OOH services are mostly general practitioners (GPs) who take regular courses in emergency medicine. Unlike in many other countries, in Norway, on-call physicians are usually in contact with emergency patients before hospitalization. However, patients in time-critical situations can be transported directly to a hospital by an EMT without consulting a physician, but rather in cooperation with the EMCC. A recent study of acute admissions to Norwegian hospitals revealed that approximately 65% were referred by GPs or on-call physicians, while 35% were directly admitted [3].

In Norway, HEMS is dispatched to cases of severe illness or trauma with an anticipated need for treatment or supervision by an anesthesiologist. In addition to helicopters, this service includes rapid response cars used for missions near the base or when the helicopter is unavailable, e.g. in bad weather [4].

Cooperation between prehospital services is vital to ensure that patients receive the correct level of care. Over-triage is to some extent necessary and acceptable to ensure adequate care for patients requiring an anesthesiologist or on-call physician. Retrospective evaluation shows that some missions could have been handled with less use of resources. In addition to the accepted over-triage, different approach regarding call-outs from OOH services can contribute to unintentionally increased HEMS usage when on-call physicians are unavailable.

One previous study reported the decreasing involvement of on-call physicians in acute medical situations in one HEMS area in Norway [5]. Another study revealed no increase of HEMS use after an organizational change in an OOH district, which led to fewer physicians on-call and a larger response area for these physicians [6]. There is limited knowledge regarding HEMS usage in areas where the on-call physician do not respond with a call-out. For future service planning, and to ensure correct allocation of resources, it is of great interest to evaluate the use of HEMS in OOH districts that apply different approaches regarding call-outs from local primary care physicians.

Municipalities in Norway differ in their organization of OOH services. Some municipalities have a policy of calling out in almost all emergencies, while others lack the infrastructure to fulfill the demand, e.g. due to lack of rapid response cars for on-call physicians [7]. Reports indicate that on-call physicians have been less available during the last decade compared to in previous years [8–10], and HEMS may be used in missions that could alternatively be handled by OOH services.

The present study investigates how different availability of an on-call physician to attend emergency patients at site (call-out) impacted requests and use of HEMS. We also aimed to explore differences between patients encountered by HEMS and OOH on-call physicians.

## Methods

### Geographical setting and organization of services

The city of Bergen is located on the west coast of Norway, has about 300,000 inhabitants (2020), and spans an area of 445 km^2^. The OOH service is organized with one large casualty clinic that is open 24/7. There are also three smaller casualty clinics in the suburban areas, which are open from 4–10 pm on weekdays and during daytime hours on weekends. Until November 2018, the OOH service was not able to perform call-outs. Near Bergen, there are two smaller municipalities, Os and Samnanger, where a total of 23,455 inhabitants (2019) live in an area of 409 km^2^. This region has an intermunicipal OOH service with one casualty clinic, and has a rapid response car available for call-outs.

Haukeland University Hospital (HUS) is the nearest hospital to all three municipalities. The driving distance to HUS is approximately 5 min from the Bergen OOH casualty clinic, and approximately 30 min from the intermunicipal casualty clinic at Os. HEMS Bergen covers the three municipalities, and is located near HUS and a two-minute drive from the casualty clinic in Bergen. The majority of the inhabitants of Bergen city can be reached faster by the rapid response car than a helicopter. The ground ambulance service in Bergen municipality has four stations. On weekdays, 13 ambulances are available during the daytime and 5 in the evening/night. Eight ambulances are available on weekends. In the municipalities Os and Samnanger, one ambulance is available 24/7.

### Design

The study was a prospective, observational study. Until November 2018, the OOH service in Bergen had no car available for call-outs in emergencies. Acute medical situations were handled by ambulance workers, without physician involvement in the majority of cases. In less severe situations, on-call physicians were contacted by telephone, or the patient was transported to the casualty clinic. In more severe situations, HEMS Bergen was requested to assist the ambulance workers. In the municipalities Os and Samnanger, the OOH on-call physician was alerted in all acute medical situations, and most often responded with a call-out. In severe cases, HEMS was requested together with ground ambulances and the on-call physician.

The three municipalities are served by the same EMCC, HEMS base, and hospital, but had different approaches to call-outs from the OOH service in acute medical situations. They also differed in their distances to HEMS Bergen. No changes occurred in the prehospital system in this area during the period of data collection for the present study.

### Data material

Our analysis included all acute medical missions, outside the hospital, with an on-site physician, in the three municipalities during the 13-month period from November 1^st^ 2017 to November 30^th^ 2018. For all missions in Bergen, we used data from HEMS Bergen, registered in the database “Airdoc”. The registered data included patient data (age, gender, International Classification of Diseases code (ICD-10), National Advisory Committee for Aeronautics (NACA) score, symptoms, clinical findings, and treatment provided) and operational data regarding each mission with patient encounter. All registered missions from the OOH service in Os/Samnanger (hereafter called “rural OOH-service”) with an on-site on-call physician were registered using an iPad with a digital form including the date, symptoms, clinical findings, treatment administered (and by whom), location, destination, NACA, and diagnosis code (ICPC-2). Data collection was planned and prospective to ensure a valid data set. A nurse at the rural OOH service continuously followed-up on missing data during the study period. Through the AMIS database, used in the EMCC, we collected the total number of acute medical missions in the municipalities.

### Data presentation and statistical analysis

Data are presented according to groups of patients within the Bergen municipality who were attended by HEMS, and patients in the rural OOH district who were attended by the on-call physician. To compare diagnostic codes from ICD-10 and ICPC-2, we categorized the diagnoses into ten predefined symptom categories [11]. Interventions and treatments were categorized into none, basic, and advanced (Table 1), where advanced interventions being performed only by anesthesiologists. It is expected that basic interventions can be performed by on-call physicians, based on personal experience and training scenarios at mandatory courses in emergency medicine. NACA scores of 5–7 were considered to represent patients with acute threat to life, and thus this score was dichotomized into 0–4 and 5–7.

**Table 1:** Categories of basic and advanced interventions used in this study

Basic
▪Chest compressions
▪Establish intravenous access
▪Establish intraosseous access
▪ECG
▪Blood glucose measurement and management
▪Prehospital thrombolysis
▪Treatment of seizures and overdoses
▪Stabilize and splint fractures
▪Stop external bleeding with compression, elevation, packing, and/or tourniquet
▪Pain treatment
▪Immobilization of trauma patient using a splinting device (e.g. SAM sling)
▪Use of other drugs available in the ground ambulance service/GP (cyklokapron, amiodarone, furosemide, Solu-Cortef, ondansetron, nitroglycerine, acetylsalicylic acid)
**Advanced**
▪Intubation/tracheostomy
▪Mechanical ventilation
▪Thoracostomy/chest drain
▪Chest compression device
▪External cardiac pacing
▪Anesthesia
▪Central venous or arterial cannulation
▪Blood products
▪Use of ultrasound or nerve blocks
▪Use of other drugs not available for the ambulance/GP
(ketamine, fentanyl, and suxamethonium chloride)

GPs are expected to perform basic interventions, whereas advanced interventions are only to be performed by an anesthesiologist

Standard descriptive analyses were performed using SPSS Statistics Version 25 IBM Corp., Armonk, NY, USA). Due to skewed data, age is presented as median with interquartile range (IQR) and compared using Mann–Whitney U-test. Fisher’s exact test and Pearsons’s Chi-square test were used for categorical variables. A *p* value of < 0.05 was considered statistically significant. Incidence is presented as rate per 1000 inhabitants per year, with the 95% confidence interval (CI).

### Ethics

The study was approved by the Regional Committee for Medical and Health Research Ethics (2017/283/REC West, Norway). Prior to analyses, the patient identification variables were deleted by the main author (DSN).

## Results

Table 2 shows that the rates of acute medical missions per inhabitants per year were similar between Bergen and the rural OOH district. The rate of HEMS requests was significantly higher in the rural OOH district compared with Bergen (*p* < 0.05). However, the rate of missions, in which HEMS attended the patients, did not significantly differ between Bergen and the rural OOH district. Table 2 also presents the numbers and rates of HEMS subcategories and types of patient transport. We found significant differences in the shares of helicopter use and rapid car missions, with the majority of helicopter missions occurring in the rural OOH district, and the majority of rapid car missions in Bergen (*p* < 0.05). OOH call-outs exclusively occurred in the rural OOH district, and were used in 66% of acute medical missions. Overall, a physician was sent to the site in 70% of acute medical missions in the rural OOH district (HEMS physician or OOH physician), compared to 4% of such incidents in Bergen (HEMS physician exclusively). HEMS served Bergen and the rural OOH district areas at rather similar rates.

**Table 2:** Acute medical missions, request for helicopter emergency medical service (HEMS) and out-of-hours (OOH) call-outs

*Variable*	**Bergen**		**Rural OOH district**		***P* value**
	*n*	*Rate (CI)*	*n*	*Rate (CI)*	
Acute medical missions	9176	30 (29–31)	744	29 (27–32)	0.61
HEMS requested	513	1.7 (1.5–1.8)	62	2.4 (1.8–3.1)	< 0.05
HEMS cancelled	234	0.8 (0.7–0.9)	32	1.3 (0.8–1.7)	0.42
HEMS encountered	279	0.9 (0.8–1.0)	30	1.2 (0.8–1.2)	0.15
Helicopter missions	30	0.1 (0.1–0.1)	24	1.0 (0.6–1.4)	< 0.05
Rapid car missions	249	0.9 (0.8–1.0)	6	0.3 (0.1–0.5)	< 0.05
OOH call-outs	0	0 (0)	493	20 (18–21)	< 0.05

Numbers and rates (per 1000 inhabitants per year) of acute medical missions, request for and subgroups of helicopter emergency medical service (HEMS) responses, and out-of-hours (OOH) call-outs in the municipality Bergen and in the rural OOH district

*p* value analyzed between rates in Bergen and in the rural OOH district

Table 3 presents demographic data regarding the patients in the rural OOH district and Bergen, according to the different services. The median age was significantly higher for the patients attended by OOH on-call physicians in the rural OOH district compared with patients attended by HEMS in both areas (*p* < 0.05). Among the patients encountered by HEMS in the rural OOH district, 80% were attended on-site, by the on-call physician.

**Table 3:** Demographic data regarding gender, mean age, medical condition, and destination categorized into three groups

	**Bergen**		**Rural OOH district**			
	**HEMS**		**HEMS**		**OOH**	
	*n*	%	*n*	%	*n*	%
*Gender*						
Female	96	34.4	10	33.3	209	42.4
Male	183	65.6	20	66.7	273	55.4
Missing	0	0	0	0	11	2.2
Total	279		30	100.0	493	
* Age*	*Median*	*IQR*	*Median*	*IQR*	*Median*	*IQR*
Female	53.0	27–70	47.0	12–51	54.0	33–77
Male	51.0	29–70	57.0	32–71	59.0	34–74
Total	51.0	29–70	50.0	27–62	58.0	34–75
* Medical condition*	*n*	*%*	*n*	*%*	*n*	*%*
Cardiac arrest	78	28.0	7	23.3	17	3.4
Trauma	82	29.4	10	33.3	83	16.8
Breathing difficulties	15	5.4	1	3.3	45	9.1
Chest pain	5	1.8	2	6.6	104	21.1
Stroke	3	1.1	0	0.0	51	10.3
Acute neurology, e.g. stroke	28	10.0	3	10.0	60	12.2
Psychiatry, including intoxication	12	4.3	0	0.0	43	8.7
Obstetrics and childbirth	7	2.5	2	6.6	10	2.0
Infection	15	5.4	1	3.3	26	5.3
Other	30	10.8	1	3.3	49	9.9
Missing	4	1.4	1	3.3	5	1.0
	279	100.0	30	100.0	493	100.0
* Destination*	*n*	*%*	*n*	*%*	*n*	*%*
Treated on site	0	0.0	0	0.0	65	13.2
Dead on site	43	15.4	7	23.3	17	3.4
Casualty clinic	27	9.7	3	10.0	42	8.5
Hospital	207	74.2	20	66.7	329	66.7
Other	2	0.7	0	0.0	6	1.2
Missing	0	0.0	0	0.0	34	6.9
	279	100.0	30	100.0	493	100.0

*HEMS* Helicopter emergency medical service, *OOH* Out-of-hours, IQR inter-quartile range

We identified significantly different patterns of symptom diagnoses between HEMS and OOH services. Cardiac arrest and trauma were the major symptom categories among patients encountered by HEMS in both Bergen and the rural municipalities (57.4% and 56.6%, respectively), while these two categories represented only 20.2% of patients attended by OOH services in the rural OOH district. Chest pain was the most frequent reason for an OOH call-out in the rural OOH-district (21.1%).

The hospital was the final destination for 74.2% of the patients encountered by HEMS in Bergen, and 66.7% of the patients encountered by on-call physicians in the rural OOH-district (*p* < 0.05). Among patients in the rural OOH district, the hospitalization rate was the same between those attended by HEMS compared to those attended by the on-call physician alone (66.7%). A NACA score of 7 (death) was more frequent among HEMS patients.

**Fig. 1:**
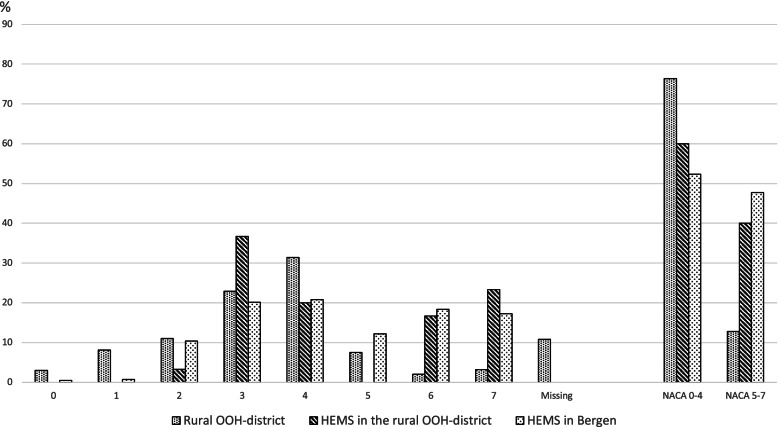
Distribution of NACA scores. Distribution of NACA scores in primary missions with patient encounters by an on-call physician in the rural out-of-hours (OOH) district, helicopter emergency medical service (HEMS) in the rural OOH district, and HEMS in Bergen

Figure 1 presents the distributions of NACA scores in the OOH service and HEMS. A NACA score of 5–7 was reported in 47.7% of the HEMS patients in Bergen, compared to 40.0% of HEMS patients in the rural OOH district (*p* = 0.44). Among patients attended by an on-call physician in the rural OOH district, 12.8% had a NACA score of 5–7 (*p* < 0.001, compared with HEMS in Bergen).

Table 4 shows the usage of different interventions in each group. Advanced interventions were administered to approximately one-third of the patients attended by HEMS in both Bergen and in the rural OOH district. Of those patients, a NACA score of ≥ 5 was reported for 87.6% of the patients in Bergen and 54.5% of those in the rural OOH district. Intubation accounted for 75% of the advanced interventions in Bergen and 45.5% in the rural OOH district. Patients with cardiac arrest and trauma were most commonly administered advanced interventions: 59.6% and 24.7%, respectively, among HEMS patients in Bergen; and 27.2% and 36.3%, respectively, among HEMS patients in the rural OOH district.

**Table 4:** Level of treatment performed by physicians

	**Rural OOH district**		**HEMS in Bergen**		**HEMS in rural OOH district**	
	*n*	*%*	*n*	*%*	*n*	*%*
None	211	42.8	83	29.7	2	6.7
Basic	282	57.2	107	38.4	17	56.7
Advanced	0	0.0	89	31.9	11	36.6

Level of treatment performed by on-call physician in the rural out-of-hours (OOH) district, helicopter emergency medical service (HEMS) in Bergen, and HEMS in the rural OOH district

## Discussion

The similar rates of completed HEMS missions in Bergen and in the rural OOH district indicate that the decision to use HEMS was not affected by the type of transport, or the distance between the patient and HEMS base. Neither did the attendance of an on-call physician from the OOH service have impact on the use of HEMS. The rate of acute medical missions was also rather similar between the municipalities, indicating that the EMCC assigns the same level of urgency regardless of patient location. HEMS was performing advanced interventions to the same amount of patients in both areas and indicating that patients in Bergen and the rural OOH district have same degree of severity and need for advanced treatment performed by HEMS.

A systematic review concluded that HEMS use is region-specific, and that dispatch criteria should be adjusted to the specific prehospital system [12]. In Norway, HEMS response requires a medical indication and acceptance from the HEMS physician. If the EMCC had requested HEMS more frequently in Bergen compared with the rural OOH district, we would expect a higher number of cancelled requests in Bergen since the rates of completed missions were similar. However, our data indicated the opposite trend, with a higher rate of cancelled requests in the rural OOH district compared with Bergen—although this difference was not statistically significant. It is possible that the EMCC may request HEMS at an earlier stage in the rural municipalities, due to the increased distance and response time compared to missions in Bergen. Additionally, an on-call physician may already have attended patients in the rural OOH district, and concluded that there was no medical indication for HEMS. Finally, sometimes a patient may be less critically ill than expected, resulting in mission cancellation.

Prehospital services staffed with anesthesiologists are used worldwide, but comparison among European countries reveals large variations in the availability of helicopters for medical emergencies [13]. The systems used by Scandinavian countries are similar in many ways, but also differ in the volume of patient encounters, service areas, and time variables [14]. Compared with Norway and Finland, Denmark and Sweden have higher volumes of patient encounters by prehospital services. In Denmark, rapid response cars are staffed with anesthesiologists, and GPs do not play the same role in acute emergencies compared with Norway. While it is not necessarily a goal to ensure similar services across the borders, it is useful to exchange knowledge about how organizational differences and changes affect other prehospital services, which can contribute to improving resource use and allocation.

There are debates regarding the benefits of HEMS use. A Cochrane review concluded that it remains unclear which elements of HEMS service benefit trauma patients: rapid transport and/or advanced interventions [15]. Patients with NACA scores of 4–6 are thought to have better outcomes when attended by HEMS [16]. However, the validity of the NACA score has not been thoroughly examined, and one study revealed large differences between individual raters and references in some clinical cases [17]. The “First Hour Quintet” (cardiac arrest, respiratory failure, trauma, acute coronary syndrome, and stroke) are critical conditions with great importance in prehospital emergency care [18], and are conditions for which HEMS can be indicated. Patients encountered by HEMS frequently receive advanced interventions, especially airway management, such as intubation [19]. As isolated variables, NACA score, clinical condition, and use of advanced interventions are not sufficient to indicate whether HEMS is necessary; however, these measures can be used together to determine the need for HEMS, and are useful for comparison between different services.

In the present study, the NACA scores among HEMS patients were similar between patients in Bergen vs. the rural OOH district, indicating that the lack of on-call physicians on site in Bergen did not lower the severity threshold for HEMS use in this area. Comparing NACA scores between call-outs from the OOH service and HEMS revealed significantly higher NACA scores among HEMS patients. This illustrates that medical emergencies represent a continuum from moderate to life-threatening situations, and that the OOH services in Norway handle a majority of patients with mild and moderate symptoms, while HEMS has expertise in treating patients with life-threatening conditions. Nationally, among patients treated within the OOH services in 2018, 7.7% have an acute and potentially life-threatening situation [20], while 62% of patients attended by HEMS have a NACA score of 4–7 [21]. This is similar to findings regarding NACA score among HEMS patients in Denmark [22]. Still, many of the patients attended by HEMS in Bergen and the rural OOH district had a NACA score of ≤ 3. This reflects the difficulties faced by EMCC operators when performing triage with limited information about the patients. In Norway, an over-triage of requesting HEMS is accepted, to reduce late arrivals and the potential negative influence on patient outcomes [16].

With regards to symptom categories, the HEMS group showed significantly higher rates of cardiac arrest and trauma compared with the OOH service in the rural OOH district, while stroke and chest pain were more frequent in the rural OOH district. Previous findings suggest that HEMS may improve survival in cases of cardiac arrest outside of the hospital, primarily after return of spontaneous circulation (ROSC) [23]. Although cardiac arrest is a life-threatening situation, HEMS requests may be cancelled based on further information about the onset time of bystander CPR, comorbidities, and clinical findings; therefore, not all patients with cardiac arrest were attended by HEMS in the rural OOH district. It is likely that HEMS use was more commonly indicated when it was expected to promote a better health outcome compared with ground ambulance and/or on-call physicians alone. Stroke is a time-critical condition that benefits from rapid transport to hospital. The relatively short travel distance to the hospital from the rural OOH district can explain why few patients with symptoms of stroke were encountered by HEMS.

Advanced interventions were most commonly performed for patients with NACA scores of ≥ 5 in Bergen. Retrospective evaluation reveals that advanced interventions can sometimes have poor effects—for example, intubation of a patient who ultimately has a NACA score of 7 (death) would not have the intended effect, but should not be considered an unnecessary intervention, as it is difficult to predict which patients will benefit from resuscitation. The fact that advanced interventions are mostly used in cases with cardiac arrest and trauma with a NACA score ≥ 5 indicates a correlation between severity and the need for HEMS.

Our present results showed a significantly lower rate of hospitalized patients who were attended by an on-call physician in the rural OOH district, compared with those attended by HEMS in Bergen. This is probably because the on-call physician attended patients with all grades of severity, and also due to the effect of having the on-call physician on site. Among patients encountered in the rural OOH district, the hospitalization rate was the same between those attended by HEMS compared to the on-call physician. Although the NACA scores were lower in the group attended by the on-call physician, equal proportions of the patients required admission to the hospital.

The role of on-site attendance by an on-call primary care physician is uncertain [24]. The presence of on-call primary care OOH physicians in medical emergencies in Bergen may be less important, since the ambulance service in Bergen has short transport distances to both the hospital and the OOH casualty clinic. In Norway, EMCC dispatches prehospital resources based on the limited information given by the caller and the potential severity, using a criteria-based triage system called the Norwegian Index for medical emergency assistance (Index) [25]. When warranted, HEMS is requested in addition to ground ambulances and on-call physicians, rather than as a replacement. In severe emergencies, multiple resources are often needed. Notably, in 2019, HEMS requests were cancelled in 14.2% of missions due to concurrencies, bad weather, or technical reasons [26]. Our present results demonstrate this resource allocation within the rural OOH district, where 80% of the HEMS missions also had an on-call physician at the site. The overlap and cooperation between different services is a strength of the prehospital system in Norway. Further research should investigate which patients benefit from attendance by an on-site physician, and how dispatch criteria can be more accurate.

### Strengths and limitations

The two OHH services compared in our study had different abilities to call-out, and no major changes occurred during the study period. The inhabitants of the municipalities were all served by the same hospital, EMCC, and HEMS base. Our analyses included all data from HEMS Bergen in the three municipalities, and all registered call-outs from the OOH service in the rural OOH district. However, there are several differences between these areas. The city of Bergen is much larger than the municipality center of Os and Samnanger, which may correspond to increased numbers of intoxications and traumas. Furthermore, the data were from one EMCC area, and more robust data could have been obtained through multicenter data collection. Notably, HEMS attended only 30 patients in the rural OOH district. Nevertheless, our results are likely generalizable to similar geographical areas in Norway. Our present study did not include data regarding outcome among the hospitalized patients. which could have given knowledge if treatment and level of care had impact on survival.

## Conclusions

Our results did not show different use of HEMS between the two compared OOH districts; however, the rate of HEMS requests was significantly higher in the rural OOH district. Additionally, NACA scores were significantly lower among patients attended by on-call physicians alone compared to those attended by HEMS. Use of advanced interventions did not differ between patients attended by HEMS in urban vs. rural OOH districts. Overall, the threshold for HEMS use seems to be independent of the availability of on-call primary care physicians, and we found no reasons to recommend a change in the current policy for accepting missions in HEMS.

## Data Availability

The datasets used and analyzed during the current study are available from the corresponding author on reasonable request.

## Abbreviations

CI: Confidence interval
EMCC: Emergency Medical Communication Center
EMT: Emergency Medical Technicians
GP: General Practitioner
HEMS: Helicopter Emergency Medical Service
HUS: Haukeland University Hospital
ICD-10: International Classification of Diseases code
ICPC-2: International Classification of Primary Care
Index: Norwegian Index for medical emergency assistance
LEMC: Local Emergency Medical Communication Centers
NACA: National Advisory Committee for Aeronautics
OOH: Primary care out-of-hours service
Rural OOH-service: OOH service in the municipalities Os and Samnanger
